# The Women Neuroscientists in the Cajal School

**DOI:** 10.3389/fnana.2019.00072

**Published:** 2019-07-16

**Authors:** Elena Giné, Carmen Martínez, Carmen Sanz, Cristina Nombela, Fernando de Castro

**Affiliations:** ^1^Sección Departamental Biología Celular, Facultad de Medicina, Universidad Complutense de Madrid, Madrid, Spain; ^2^Department of Biological and Health Psychology, Universidad Autónoma de Madrid, Madrid, Spain; ^3^Fundación para la Investigación Biomedica del Hospital Clínico San Carlos, Hospital Clínico San Carlos, Madrid, Spain; ^4^Grupo de Neurobiología del Desarrollo-GNDe, Instituto Cajal (CSIC), Madrid, Spain

**Keywords:** history of neuroscience, Spanish Neurological School, Laura Forster, Tello, de Castro, Lafora, female neuroscientists, pioneer female scientists

## Abstract

At the beginning of the 20th century, in view of the growing international recognition of Santiago Ramón y Cajal, the Spanish authorities took some important steps to support Cajal’s scientific work. This recognition peaked in 1906, when Camillo Golgi and Santiago Ramón y Cajal shared the Nobel Prize in Physiology or Medicine. The Spanish government provided Cajal a state-of-the-art laboratory in Madrid to allow him to continue with his research and they funded salaries to pay his first tenured collaborators, the number of which increased further after the creation of the *Junta para Ampliación de Estudios (JAE)*. The *JAE* was an organism set up to help promising researchers develop their careers in different ways, thereby contributing to the development of science in Spain. Although largely forgotten or relatively unknown, there has been a recent revival in the recognition of the school that developed around Cajal, collectively referred to as the Spanish Neurological School (or colloquially, as the Cajal School or School of Madrid). Almost all Cajal’s collaborators were men, although a limited number of female scientists spent part of their careers at the heart of the Cajal School. Here we discuss these women and their work in the laboratory in Madrid. We have tracked the careers of Laura Forster (from Australia/United Kingdom), Manuela Serra, María Soledad Ruiz-Capillas and María Luisa Herreros (all Spanish), through their scientific publications, both in the journal founded by Cajal and elsewhere, and from other documentary sources. To complete the picture, we also outline the careers of other secondary figures that contributed to the production and running of Cajal’s laboratory in Madrid. We show here that the dawn of Spanish neuroscience included a number of contributions from female researchers who to date, have received little recognition.

## Introduction

When the neuropsychiatrist Luis Simarro (1851–1921) introduced Santiago Ramón y Cajal (1852-1934) to the Golgi method to stain neural structures, it would have been impossible to predict how fruitful this was to be in the hands of the young Spanish university professor. In a historically brief but intense period, Cajal studied almost all the structures of the nervous system in different species and his productivity at that time is still difficult to conceive. Yet most importantly, through these studies he formulated the so-called “neuron doctrine^1^” and the fundamental “law on the dynamic polarization of neurons” (Ramón y Cajal, 1917; de Castro, 1981, 2019b; Shepherd, 1991; De Carlos and Pedraza, 2014). At the dawn of the 20th century, Santiago Ramón y Cajal deserved the international reputation he received for his work on the Anatomy and Histology of the nervous system. At this point he was the paladin of the “neuron doctrine,” which proposed that neural tissue is formed of individual cells –or neurons- and not of syncytial networks. Nevertheless, most of the researchers active in the field at that time were still open or cryptic “reticularists” (Shepherd, 1991; de Castro, 2019a,b). The prestige of Cajal was boosted by the award of the Moscow International Prize (1900), the Helmholtz Medal from the German Imperial Leopoldina Academia (1905) and the still recently founded Nobel Prize in Physiology or Medicine (1906), sharing the latter with maybe the most visible leader of the reticularists, the great Italian histologist Camillo Golgi (1843–1926). However, Cajal’s scientific production was particularly remarkable in the 19th century, along which Spain lost its empire (in the period between 1815 and 1898: de Castro, 2019a,b).

**FIGURE 1:**
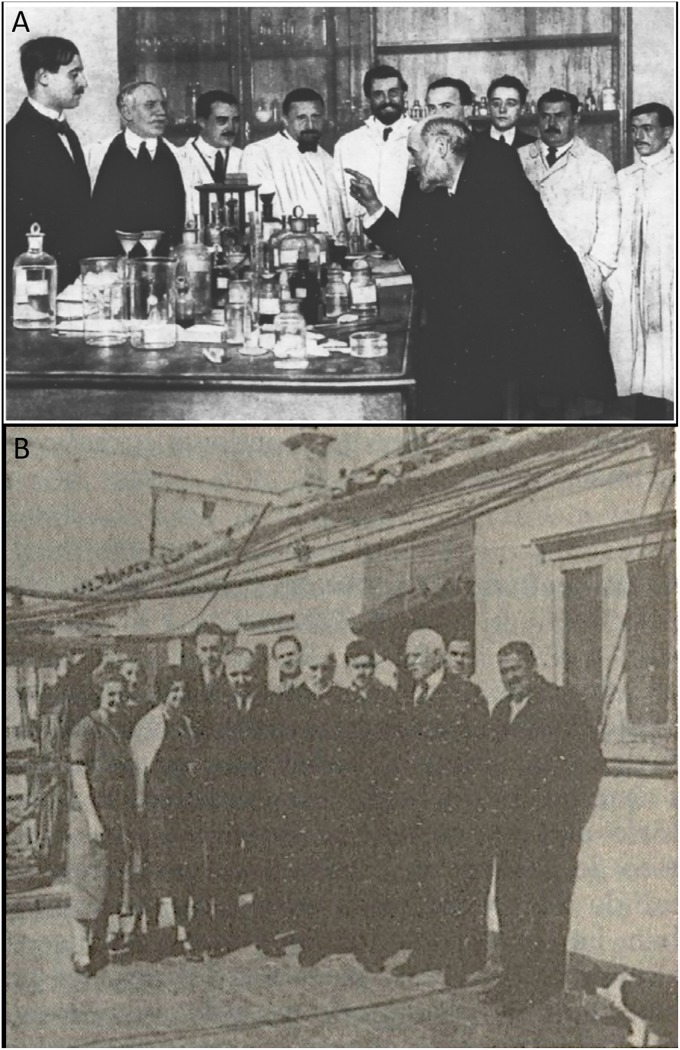
The Spanish Neurological School. **(A)** Famous photograph published in No. 56 of the journal *La Esfera* (Madrid, Spain), January 24th, 1915. From left to right: Gonzalo R. Lafora, Domingo Sánchez, José Miguel Sacristán, Miguel Gayarre, Nicolás Achúcarro, Santiago Ramón y Cajal (in teaching pose, indicating with his right hand), Luis Rodríguez Illera, Juan de Dios Sacristán, Tomás García de la Torre (concierge at the *Instituto Cajal*) and Jerónimo (laboratory assistant). **(B)** The picture was taken on the roof of the *Laboratorio de Investigaciones Biológicas*, at 13 Paseo de Atocha (Madrid, Spain). On the left of the group there are three women vaguely identified as “preparadoras” (the one in the middle is Carmen Serra –see text for details), then toward the right: Fernando de Castro, Jorge Francisco Tello, an unidentified man, Cajal, another unidentified person, Domingo Sánchez, Luis Calderón (to become a famous odontologist) and Tomás García de la Torre (the concierge who was very close to Cajal from their years during the war in Cuba). This picture was originally published in de Castro (1981), and the original belongs to the *Archivo Fernando de Castro* (Censo Guía de Archivos de España e Iberoamérica #ES.28079.AFC; Madrid, Spain) that was included by *UNESCO* in the Memory of the World International Register of the Human Heritage in 2017, as “Archives of Santiago Ramón y Cajal and the Spanish Neurohistological School” (http://www.unesco.org/new/en/communication-and-information/memory-of-the-world/register/full-list-of-registered-heritage/registered-heritage-page-1/archives-of-santiago-ramon-y-cajal-and-the-spanish-neurohistological-school/).

This international academic recognition of Cajal drove the Spanish authorities to adopt measures to support the acclaimed neuroscientist at the zenith of his career. As such, the King of Spain, Alfonso 13 (1886–1941), and the Prime Minister, Francisco Silvela (1843–1905), convinced the Spanish government to establish and fully furbish a modern Histology laboratory for Cajal in 1901 (De Carlos and Pedraza, 2014; de Castro, 2019a,b). The first collaborator recruited by Cajal to the laboratory in 1902 was Jorge Francisco Tello (1880–1958) and subsequently, the number of collaborators multiplied with the foundation of the *Junta para Ampliación de Estudios-JAE* (Council for Extended Studies), the presidency of which was entrusted almost immediately to Cajal himself (Figure 1A: Ramón y Cajal, 1917). The *JAE* was very effective in sponsoring the visits of promising Spanish students to prestigious laboratories abroad and on their return to Spain, they were encouraged to use their newly acquired skills and knowledge to the general benefit and progress of the nation. As well as the issues related to the experimental sciences and technologies, the *JAE* also covered the areas of Arts and Humanities (Caballero Garrido and Azcuénaga Cavia, 2010). Some of the young researchers funded were especially brilliant and they gave continuity to the titanic efforts of their *Maestro*, for example: Nicolás Achúcarro (1880–1918) incorporated neuropathology as a new research line in Cajal’s laboratory; Pío Río-Hortega (1882–1945) identified two of the four classic cell types that make up the CNS (oligodendrocytes and microglia); Fernando de Castro (1896–1968) unraveled the innervation of the carotid region and identified the first chemoreceptors in the carotid body; and Rafael Lorente de Nó (1902–1990), among other achievements, described the organization of the audio-vestibular system and he was the first to suggest the columnar organization of the brain (de Castro, 1981, 2019b; De Carlos and Pedraza, 2014; de Castro and Merchán, 2016). Together, these scientists became known as the Spanish Neurological (or Neurohistological) School, and more colloquially, the School of Madrid or directly the School of Cajal. When Santiago Ramón y Cajal received the Echegaray Medal from the *Royal Spanish National Academy of Physics, Exact and Natural Sciences* (1922), he listed all the members of the School (Table 1), which included two women, Laura Forster and Manuela Serra, both of whom were also mentioned in an article in the general press (Pérez, 1929). These were the first two women to develop their scientific potential in the School while Cajal was still fully active. Here, we also consider María Soledad Ruiz-Capillas and María Luisa Herreros, two more women who worked at the *Instituto Cajal^2^* between the late 1920s and mid 1940s with Gonzalo R. Lafora and Fernando de Castro, respectively. Neuroscience was important to these women and they made interesting contributions that deserve this delayed recognition. We complete our study by mentioning some important women who worked as scientific illustrators in the laboratory (Figure 1B). We have included in the present work all the biographical and scientific data we were aware of regarding these women.

**Table 1:** The School of Cajal, as he himself defined it in 1922.

*(when awarded with the Echegaray Medal, by the Spanish Royal Academy of Sciences)*
**Pedro Ramón y Cajal (1894–1918)**
**Claudio Sala i Pons (1892–1994)**
**Carlos Calleja y Borja-Tarriús (1893–1897)**
**Isidoro Lavilla.** Assistant at the laboratory of Histology (1887–1997)
**Ramón Terrazas** (1896–1897) **Tomás Blanes Viale** (1898) **Federico Olóriz Ortega** (1897) **Jules Havet** (1898–1916)
**Eduardo del Río Lara (1900–1910)**
**Rafael Forns (1903)**
**Jorge Francisco Tello Muñoz** (1903–1921) **Domingo Sánchez Sánchez** (1904–1920) **Manuel Márquez Rodríguez** (1898–1901) **Gonzalo R. Lafora** (1910–1916)
**Sánchez y Sánchez (1916–1919)**
**Fernando de Castro Rodríguez (1916–1922)**
**Nicolás Achúcarro Lund** (1911–1915) **José Miguel Sacristán** (1912–1913) **Luis Calandre Ibáñez** (1913)
**Miguel Gayarre Espinel** (1912–1914) **Pío del Rio-Hortega** (1913–1922) **Jorge Ramón Fañañás** (1912–1918) **Galo Leóz Ortín** (1912–1913)
**Lorenzo Ruiz de Arcaute** (1912–1913)
**Laura Forster** (1911) ←←
**Rafael Lorente de Nó** (1920–1922)
**Manuela Serra** (1921) ←←
**Mariano Górriz (1921)**
**José $M^{\underline{a}}$ Villaverde y Larraz** (1920–1921)


*This list conserves the order in which Cajal wrote it. In brackets, the years that Cajal considered each collaborator worked with him (obviously, up to 1922). This list was included in the 3rd edition of Cajal’s memories (Ramón y Cajal, 1923).*

## Laura Forster

**FIGURE 2:**
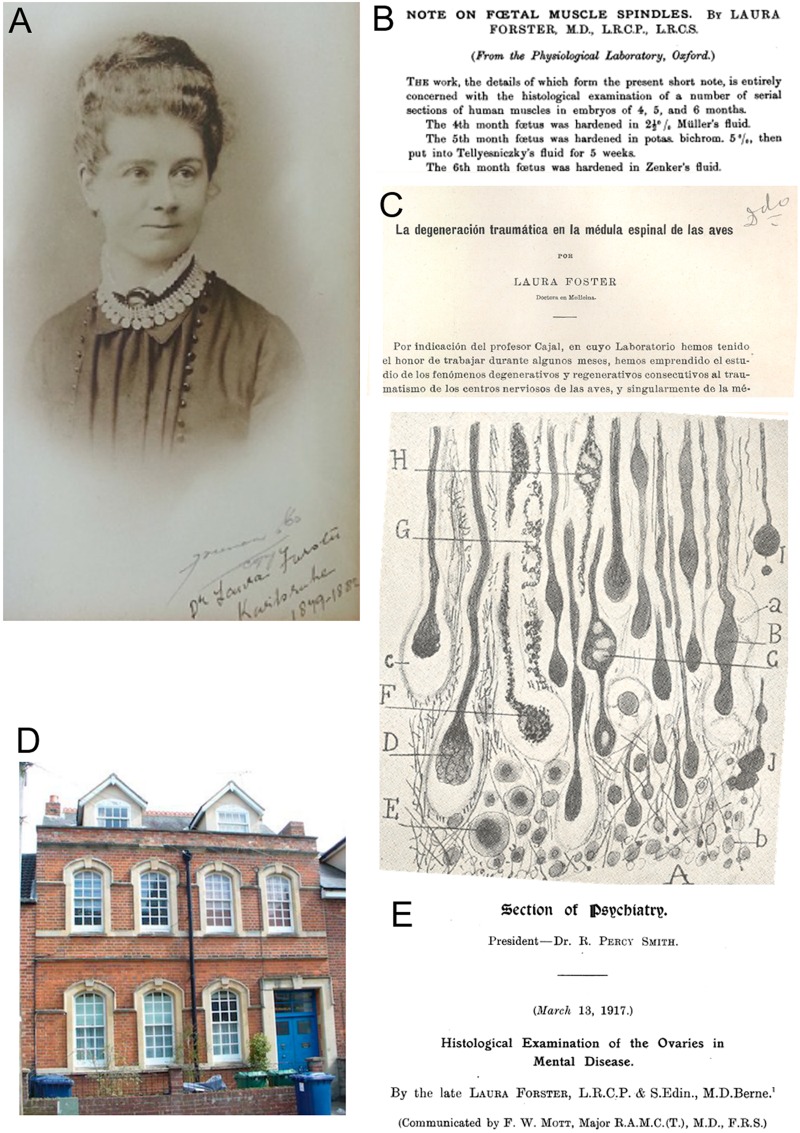
Dr. Laura Forster. **(A)** Portrait of Laura Forster in her early twenties, signed in Karlsruhe (Germany) and dated between 1879 and 1884. Source: https://en.wikipedia.org/wiki/Laura_Forster). **(B)** Publication by Laura Forster from the *University of Oxford* (Forster, 1902). **(C)** Publication by Laura Forster (wrongly written “Foster”) from Cajal’s laboratory (Forster, 1911). On the front page (upper part) there is a brief introduction in Spanish, *“by indication of professor Cajal, in whose laboratory I had the honour to work during some months”*. At the bottom, Figure 3 of the cited work produced in Madrid, showing the proximal edge of a pigeon’s sectioned spinal cord (*A*: lesion; *B,D,I,J*: engrossed axons after section; *F–H*: individual axons). **(D)** Former *Cutler Boulter Dispensary* and *Russian Orthodox Church*, at Oxford (4, Marston St) where Dr. Forster worked before going to the Balkan wars and the Ist World War. **(E)** Title of the posthumous paper by Laura Forster, communicated by F.W. Mott in her absence.

Laura Elizabeth Forster (1858–1917; Figure 2A) was born in a suburb of Sydney (Australia), the fifth of the six children of Eliza Wall and her husband, the politician William Forster (landowner and poet), a member of the *New South Wales Parliament* from 1856 to 1880 and Premier of New South Wales during 1850–1860, subsequently occupying different portfolios. Her mother died when she was a little girl (1862) and her father then married Maud Edwards, adding five more children to the family. When Mr. Forster died (1882), Laura moved to England in the company of her stepmother and one of her half-sisters. Initially educated in Australia, in 1887 Laura Forster entered the University of Bern (Switzerland) as a medical student, receiving her M.D. in 1894. There, she worked for 6 years at the *Institute of Pathology*, devoting her research to the study of muscle spindle fibers. She later published her first scientific paper on these structures when in Oxford, focusing on their development in human fetuses between 4 and 6 months of gestation (Forster, 1902; Figure 2B). In 1895 Laura Forster (M.D.) received her certificate allowing her to work as a GP in the United Kingdom (see page 34 in: “Registered during the Year 1894: The General Council of Medical Education and Registration of the United Kingdom, London, 1895”).

Trained as both a doctor and a nurse in Glasgow and Edinburgh, Forster then settled in Oxford (United Kingdom) to practice medicine. In 1900, she was appointed medical officer at the *Cutler Boulter Dispensary* in an East Oxford suburb. There she investigated the etiology of ovarian diseases and their effects in women with mental problems, coming into contact with the *Physiological Laboratory* at the prestigious University of Oxford. Indeed, the aforementioned paper Laura Forster expresses her gratitude to the director of this laboratory, Prof Gotch^3^, *“for kind permission to work in the Oxford Physiological Laboratory”*, and especially to Dr. Gustav Mann, Senior Demonstrator of Physiology and author of an important textbook entitled “Physiological Histology” (Mann, 1902), *“for his kind help and suggestions”*. Under the supervision of the Indian-born to a German father, Dr. Mann, Forster published a second scientific article on the histology of lymph nodes from a patient affected by tuberculosis (Forster, 1907).

The influence of Gustav Mann (experienced in histological staining) and the fact that he moved to *Tulane University* (New Orleans, United States) as Professor of Physiology in 1908, together with the recent international prizes awarded to Santiago Ramón y Cajal between 1900 and 1906, prompted Laura Forster to spend time in Cajal’s laboratory to gain a greater command of neurohistological techniques. According to Cajal and Forster, the latter worked for *“a few months”* in 1911 at the *Laboratorio de Investigaciones Biológicas* or Cajal’s laboratory (Forster, 1911; Table 1). Indeed, in the very first lines of her third scientific paper, Laura Forster declares that Santiago Ramón y Cajal suggested she focused her research in the lab on whether the degeneration of nerve fibers after traumatic lesion of the spinal cord in birds corresponded with events observed in previous studies on mammals performed by Cajal himself and others (Forster, 1911; Figure 2C). In fact, Forster’s study was the first time that neurofibrillary techniques were applied to birds for this purpose and her results demonstrated similarities with the process in mammals, although these occurred more rapidly in birds, describing both degenerative (retracted fibers with varicose “in ball” endings) and regenerative processes (fine nerve sprouts that penetrated the scar and the necrotic zone). This was the longest of her scientific papers to date and it was elegantly illustrated by 6 drawings in the style of Cajal or Achúcarro^4^, and even more curious was that the article is written entirely in Spanish. This paper is dated August 1911, from Madrid, expressing *“cordial thanks to Dr. Cajal for his amicable advice, as well as to Drs N. Achúcarro and F. Tello for the generous help that they gave me while performing this work^5^”* (Forster, 1911). We should highlight here that Nicolás Achúcarro, who joined Cajal’s laboratory in 1910, was the first member of the Spanish Neurological School fully devoted to study the pathology of the nervous system, while Francisco Tello spent part of 1911 as a *JAE* fellow in Germany training in Pathology and Bacteriology (de Castro, 1981). Cajal cited the work carried out by Laura Forster’s in his laboratory at least three times (Ramón y Cajal, 1913, 1914, 1917). A decade after Forster’s publication, one of the main and youngest direct disciples of Cajal, Rafael Lorente de Nó, continued to study the degeneration-regeneration in the spinal cord of non- mammalian embryos, along with Manuela Serra (see below). In this case the work was carried out on Amphibians, the study of Laura Forster forming the cornerstone of their work (Lorente de Nó, 1921; Serra, 1921).

The career of Laura Forster underwent a drastic turn in 1912 and when the First Balkan War was declared, she traveled to Epirus to enlist as a nurse, since women couldn’t serve as physicians at the war front. From that moment onward, the life of Laura Forster is linked to war. Immediately after the outbreak of the Ist World War she joined the *British Red Cross* and worked at the *British Field Hospital* in Antwerp (Belgium), becoming the first female Australian doctor to assist in the wartime medical effort, although as a woman, she was again not allowed to enlist in the *Allied Medical Corps*. After a short time working in Northern France Laura was sent to Russia where she volunteered as a surgeon at the largest hospital in Petrograd (currently, St. Petersburg). She remained there for several months after the Autumn of 1915, working *“very happily with the Russian doctors, without need of an interpreter*” (Obituaries Australia, 1917). She then joined the *Russian Red Cross* and served in the Caucasus and Erzurum (Turkey), supervising a 150 bed, infectious diseases campaign hospital in the middle of a typhus epidemic during the summer of 1916. Her final destination was a hospital in Zalishchyky, in the Galicia region (Russia), just 30 miles away from the front and attached first to the *9th* and after to the *7th Army* (General Aleksei Brusilov). That was one of the five hospitals in the region operated by the *National Union of Women’s Suffrage Societies* (United Kingdom), where thousands of soldiers and especially civilian refugees were treated for typhoid, scarlet fever, dysentery and different types of war wounds and traumatic lesions. The exhausting work, frequent bombardments and the exposure to infectious sick people seriously affected the health of Laura Forster and she died on February 11th, 1917, and was buried in Zalishchyky under Russian Orthodox rites.

Dr. Frederick W. Mott communicated to the *Royal Society* the last of Forster’s findings from her work at the *Pathology Laboratory* at the *Claybury Asylum* (London, United Kingdom; Figure 2D) and that were published posthumously in March 1917. This paper, illustrated with eight microphotographs taken from histological slides, compiled the results from the ovaries of 100 deceased women with different types of mental diseases *(“dementia praecox, mania, melancholia, general paralysis of the insane, epilepsy and imbecility”*), all collected at the asylum, at the *Charing Cross Hospital* and *London Hospital* (both in London, United Kingdom), and at the London County Asylums (Long Grove, Hanwell, Colney Hatch, Bexley, Horton, Manor, Canehill, Leavesden and Caterham: Forster, 1917; Figure 2E). This article was reprinted in 1918 (Forster, 1918) and it proved to be fundamental for subsequent work of Mott, 1921. It is also noteworthy that Dr. Miguel Prados Such, one Pío del Río-Hortega’s main disciples, received funding from the *JAE* to work in the laboratory of Frederick Mott until September 1921^6^ (Junta de Ampliación de Estudios, 1925), where together, they studied the histopathology of the sexual gonads in dementia praecox (Mott and Such, 1922).

We do not know if Cajal was aware of the singular life of his former collaborator and her death. Nevertheless, Laura Forster was relatively soon recognized as an icon for female physicians in Australia and the Commonwealth (Wagner, 2017).

## Manuela Serra

**FIGURE 3:**
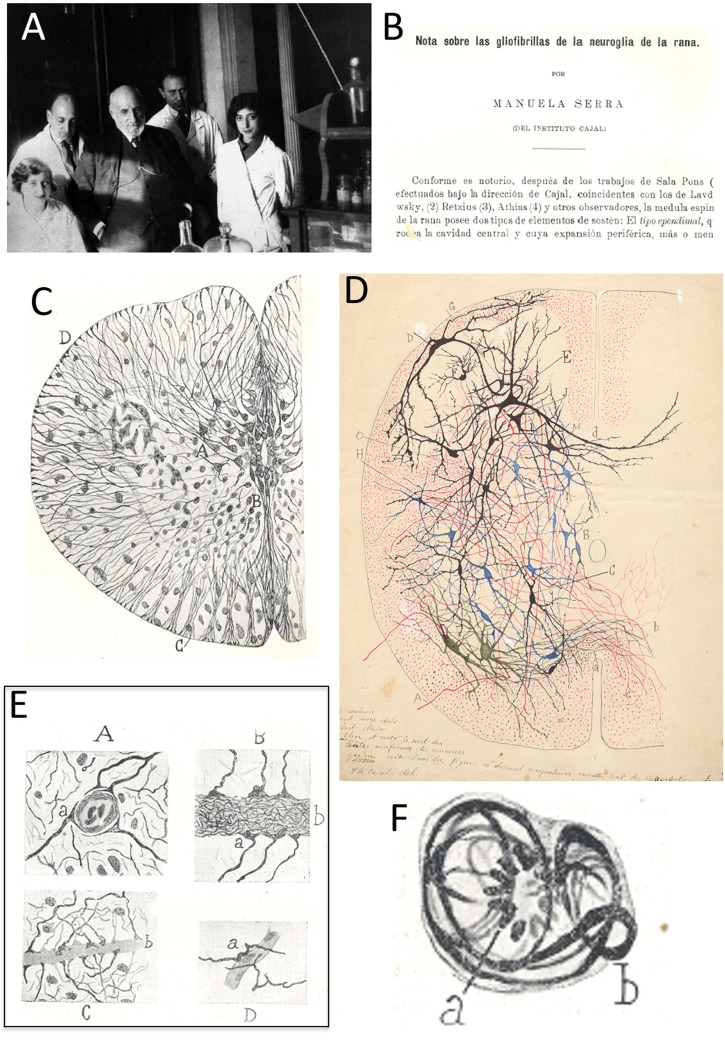
Manuela Serra. **(A)** At the *Laboratorio de Investigaciones Biológicas*, popularly known as the “Cajal Institute” (at its first site: 13 Paseo de Atocha) and from left to right, Carmen Serra (technician, sister of Manuela Serra), José $M^{\underline{a}}$ Villaverde, Santiago Ramón y Cajal, Fernando de Castro and Enriqueta “Ketty” Lewy. The presence of the latter dates the image taken to the mid-late 1920s (after 1926). **(B)** First page of the paper published by Manuela Serra in 1921, indicating she was *“from* [the] *Cajal Institute”*. **(C)** Reproduction of Figure 1 from Serra (1921), showing a transverse section of the amphibian spinal cord, including some of the main descriptions in the article, like ependymal cells with robust glio-fibrils **(A)** or subpial cones **(D)**. **(D)** Original polychrome drawing of the spinal cord by Santiago Ramón y Cajal. The differences between **C**,**D** strongly suggest that **C** (as **E,F**, see below) is an original drawing by Manuela Serra. This original drawing by Cajal (with his hand-written instruction for the publishers on the bottom-left) belongs to the *Archivo Fernando de Castro* (Censo-Guía de Archivos de España e Iberoamérica #ES.28079.AFC; Madrid, Spain), that in 2017 was considered by *UNESCO* in the Memory of the World International Register of the Human Heritage, as “Archives of Santiago Ramón y Cajal and the Spanish Neurohistological School” (http://www.unesco.org/new/en/communication-and-information/memory-of-the-world/register/full-list-of-registered-heritage/registered-heritage-page-1/archives-of-santiago-ramon-y-cajal-and-the-spanish-neurohistological-school/). **(E)** Four drawings originally comprising Figure 5 in Serra (1921), illustrating different relationships between neuroglial cells and blood vessels in the spinal cord (end-feet). **(F)** Nice drawing originally published as Figure 6 in Serra (1921), which shows a differentiated neuroglial (astrocytic) cell (with glio-fibrils) in mitotic division (phase of *“mother star”* – see footnote for the entire description of this rare but pioneer image in Spanish).

The second and only other woman listed by Cajal in his description of the School is Manuela Serra (Table 1). Very little information is available about her and we do not even have any certified photographic documentation. Like her sister Carmen, she was one of the assistants at the *Laboratorio de Investigaciones Biológicas* (Figure 3A; de Castro, 1981; Egido and Montes, 2018), and even though Manuela Serra was not a doctor or senior researcher, she was the sole author of an article published in the journal of the laboratory in 1921 (Serra, 1921; Figure 3B). This was maybe the reason why Santiago Ramón y Cajal mentioned Manuela Serra in the list of his disciples dated in 1922 and not her sister (Table 1), and she was also included as a member of the *Laboratorio de Investigaciones Biológicas* in successive years (from 1921 to 1925: Figure 4; Junta de Ampliación de Estudios, 1925).

**FIGURE 4:**
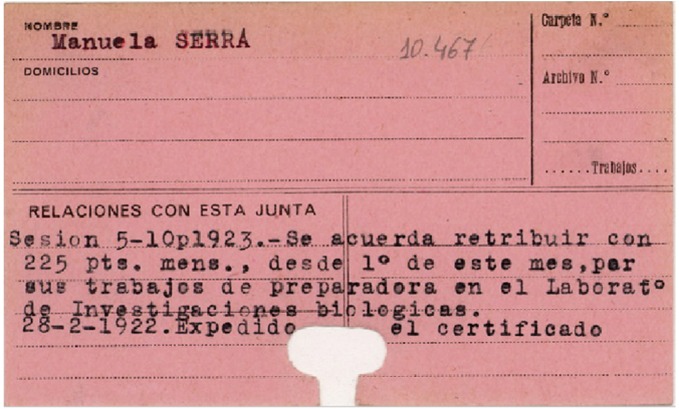
Only mention of Manuela Serra in the archives of the *Junta de Ampliación de Estudios-JAE* (Madrid, Spain). Literally, it states: *“Session 5–10 p 1923. It is agreed the retribution of 225 pesetas* [less than 1.4 euros at the current official rate of exchange] *per month, from the 1st of the month, for her work as “preparadora” at the* Laboratorio de Investigaciones Biológicas [official name of Cajal’s lab]. *Certified expended on February 2^nd^, 1922”.* This payment is undoubtedly linked to the publication by Manuela Serra, signed on January 1922 but published in the volume corresponding to 1921 (see text for details).

Partially conceived as a continuation of the initial study by Claudio Sala i Pons (Sala, 1892; observations included in Ramón y Cajal, 1909–1911), the article by Serra described the intracellular fibrils of ependymal cells and astrocytes in the spinal cord of the frog, and it was elegantly illustrated with seven figures that included a total of 10 drawings (Figure 3C,E,F). She also noticed the presence of microglia^7^ (described as “mesoglia”) in the white matter and possibly, the gray matter. Serra used the *“Cajal’s new method to color neuroglia”* (Serra, 1921), including formol-ammonium bromide in the method previously described by Max Bielschowsky (Ramón y Cajal, 1920; Ramón y Cajal and de Castro, 1933; Merchán et al., 2016). In her descriptions, Manuela Serra emphasized the sub-pial thickening of astroglial processes, as well as the perivascular end-feet previously described by Nicolás Achúcarro, Cajal himself and Fernando de Castro (Figure 3E: Ramón y Cajal, 1909–1911; Achúcarro, 1915; de Castro, 1920). Serra’s illustration of a neuroglial cell undergoing mitosis in the adult spinal cord of the frog is very interesting (Figure 3F)^8^ and while rare, it demonstrates that astrocytes can divide even when they have reached the degree of maturation where they have glio-fibrils. This phenomenon had already been noted during embryonic development by Cajal, Achúcarro, del Río-Hortega and de Castro, and in the adult CNS of both birds and mammals, as Manuela Serra summarized in her article (Serra, 1921). It is remarkable that it was not until the beginning of the 21st century that adult astrocytes were confirmed to contribute to neurogenesis in the adult CNS, the birth of new neural cells (Doetsch et al., 1999; Seri et al., 2001; for a review, see: Kriegstein and Álvarez-Buylla, 2009). Manuela Serra’s original illustrations were very high-quality, although evidently different from those of Cajal (Figure 3C,D) and those of the other masters of illustration within the Spanish Neurological School, such as Pío del Río-Hortega or Fernando de Castro (not shown). The last lines of Serra’s work are of gratitude devoted to *“our master Cajal for his guidance in the interpretation of the histological slides”*, as well as for his help with the scientific bibliography. She also thanks *“the advice of Mr Lorente de Nó, assistant at the Laboratorio de Investigaciones Biológicas”* (Serra, 1921). We want to highlight that Rafael Lorente de Nó, an important character in the Spanish Neurological School, began working as disciple of Cajal by studying the regeneration of the spinal cord in the frog larvae (Lorente de Nó, 1921), research that was contemporary to that published by Serra (1921). Undoubtedly, both the young Lorente de Nó’s studies and those of Serra were a logical continuation of the studies performed by Laura Forster in the laboratory of Cajal a decade before (see above). Curiously, the manuscript by Manuela Serra is signed and dated January 1922, while it was published in the volume of the journal from the previous year, and Cajal indicates that collaboration with Manuela Serra was in 1921 (Table 1).

## María Soledad Ruiz-Capillas

**FIGURE 5:**
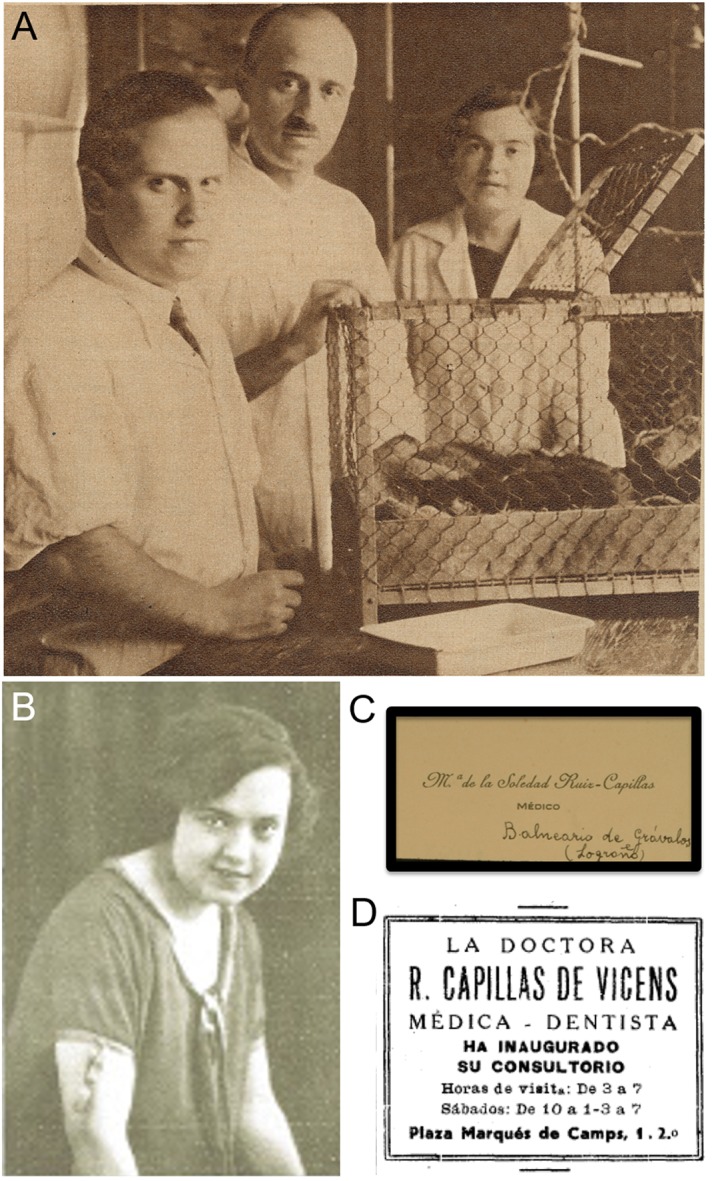
Dr. María Soledad Ruiz-Capillas. **(A)** Image of the group led by Dr. Gonzalo R. Lafora (in the middle) at the *Cajal Institute*: on the left, Dr. Julián Sanz-Ibáñez; on the right, Dr. Soledad Ruiz-Capillas (originally published in Pérez (1929) – the picture was taken by the author himself, Dr. Fernán Pérez). **(B)** Condolence card from Dr. Ruiz-Capillas after the death of Santiago Ramón y Cajal, conserved in the *Legado Cajal* (published in the present work with permission and courtesy of the *Cajal Institute, Cajal Legacy, Spanish National Research Council (CSIC)*, Madrid, Spain). **(C)** Portrait of Dr. Ruiz-Capillas used to illustrate an interview published in the magazine *Nuevo Mundo* (Madrid, 10-IV-1925). **(D)** Press announcement of the dentistry clinic of Dr. Ruiz-Capillas in Gerona (Spain), published on the same page as that of another interview referred to herein (Rodríguez-Grahit, 1935).

The first Spanish woman with a university degree that worked in Cajal’s circle was María Soledad Ruiz-Capillas, born in Toledo on February 28th 1902 to Rogelio Ruiz-Capillas, a commander in the *Spanish Army Corps of Engineers*. María Soledad was educated at the *Instituto Provincial* (Toledo), later at the *Instituto Cardenal Cisneros* (Madrid) (García Martín, 2017), and in 1917 she began to study Medicine at the *Universidad Central* (Madrid). Having successfully passed all the exams in the first 3 years of her degree in medicine with high grades, she was the best of the 73 aspirants that applied for the position as “alumno interno” at the *Beneficencia Provincial* (Madrid), which was therefore offered to her (Rodríguez-Grahit, 1935). This was a position in which the Medical students were entrusted to give patients the prescriptions determined by the physicians and they supervised the patient care given by the nurses. Once she obtained her M.D. in 1924, Dr. Ruiz-Capillas was appointed to direct the spa at Fuensanta de Gayangos (Burgos) in 1925. She was the first woman in such a position in Spain and from there, she moved to other spas at Arechavaleta (Basque Country) and then Grávalos (La Rioja – see below), always as the Director of the institution (Rodríguez-Grahit, 1935). In 1928, Dr. Ruiz-Capillas made a drastic change in her career and became part of the research group of the neuropathologist and neuropsychiatrist Gonzalo R. Lafora at the *Instituto Cajal* (nominally, *Laboratory of General Physiology*), financed by the *JAE*. Between 1928 and 1930, María Soledad Ruiz-Capillas worked under the direction of Gonzalo R. Lafora and his assistant Julián Sanz-Ibáñez, studying the neural centers involved in sleep pathologies (Figure 5A: Pérez, 1929; Junta de Ampliación de Estudios, 1931). Specifically, she collaborated in studies of the diencephalic thermal centers in the cat, sleep problems derived from infundibular and mesencephalic lesions, and how infusing diverse ionic solutions and other substances (calcium, potassium, magnesium, luminal, opioids) affected sleep, in this case employing new direct approaches to the IIIrd ventricle designed by the group (Junta de Ampliación de Estudios, 1931). Sleep problems were also studied in catatonic animal models that received Spiegel’s dual thalamic ablation. To study diencephalic and mesencephalic physiology, researchers at the General Physiology Laboratory chemically destroyed the walls of the ventricles by intraventricular injection of colored caustic solutions (turpentine with Nile blue, Müller liquid), and Dr. Ruiz-Capillas was specifically entrusted with determining the exact site of the damage, as well as with comparing the histology of the normal and damaged structures (Junta de Ampliación de Estudios, 1931). Dr. Ruiz-Capillas described the atmosphere at the institute, particularly that surrounding the *Maestro* Cajal: *“When the maestro talked to us, from behind the experimental table and in his immaculate white laboratory coat, a religious silence invaded the laboratory. All of us looked at him with the fervour that only Science can instil. His words were engraved on our brains to never be forgotten…”* (Rodríguez-Grahit, 1935). During those years, Dr. Ruiz-Capillas combined her research in Dr. Lafora’s laboratory with the study of dentistry, which she completed with outstanding academic results in 1934. The Odontology School, officially founded in 1914, allowed students to join the School after a minimum of 2 years at Medical School, and after two more years practice in Odontology and a special examination, they were awarded an official diploma as a dentist (Pardo Monedero, 2013). In the academic year 1930–1931, just 15 out of a total 405 alumni at the *Dental School of Madrid* were women.

In 1932, the *Instituto Cajal* moved from the building at No. 13 Paseo de Atocha to a new site at San Blas hill, within the Retiro Park. This initially advantageous change generated a problem of incompatibility between the new electrical installations, working from AC, and most of the scientific equipment used in the old building that worked on DC. This problem meant electrical drills, microphotography systems, exploration and surgery lamps, etc., could no longer be used and therefore, there was a substantial delay in the progress of the research into diencephalic and mesencephalic physiology (Junta de Ampliación de Estudios, 1933). Perhaps it was this inconvenience that led Dr. Ruiz-Capillas to put an end to her research in Cajal’s Laboratory and she took a position as an assistant at the Odontology Clinics in the *Carabanchel Military Hospital* (Madrid), as well as a post as director of the Grávalos spa (La Rioja), from where she sent her letter of condolences upon the death of Santiago Ramón y Cajal that is conserved in the *Legado Cajal* (Figure 5B,C).

To our knowledge, she never published a scientific paper and subsequent Lafora’s scientific communications in this field didn’t include Dr. Capilla’s as an author (Lafora and Sanz-Ibáñez, 1931a,b). In direct relationship with this publication, the newspaper *ABC* (April 12th, 1931; page 42 of the morning edition) announced that on Monday 13th at 19:00 *“Doctors Lafora and Sanz will present their personal experiences on the neural sleep centres, with fixed and cinematographic projections”* at the *Spanish Medical Chirurgical Academy* (6 Esparteros street, Madrid). Given the transcendental local elections celebrated the Sunday April 12th, prior to the presentation and their consequences (although Republican candidates received fewer total votes than the Monarchists ones, the results give rise to the resignation of the king Alphonse XIII and the advent of the IInd Spanish Republic, that was officially proclaimed in Madrid on April 14th 1931; Álvarez Tardío and Villa, 2017), we cannot confirm whether the presentation of Drs Lafora and Sanz finally took place or not, although we do know that their paper was published. Despite his immense bibliographic production (at least 247 scientific articles were published by Gonzalo R. Lafora during his lifetime), his research line on the physiology and physiopathology of sleep was particularly relevant in his career since it was the subject he chose for his acceptance speech at the Spanish National Academy of Medicine in May 14th, 1933 (López-Muñoz et al., 2009).

After some years in which there is no information available, we know that María Soledad Ruiz-Capillas opened an Odontology clinic at the beginning of 1935 in Gerona (North of Catalonia, close to the border with France) (Figure 5D), and she is considered to be the first woman working as a physician in this Spanish Province, even before Dr. Francesca Casaponsa i Suñol (1906–1990) who is currently (and wrongly) considered the first female doctor there (Ausín Hervella and Calbet Camarasa, 2010). After the Spanish Civil War, Dr. Ruiz-Capillas worked in Palma de Majorca and she ultimately died in Alicante, in 1990.

## María Luisa Herreros

María Luisa Herreros García was born in the industrial town of Torrelavega (Santander, Northern Spain) on October 3rd, 1917, where her father was the owner of a business dedicated to carpentry and marble stonemasonry, while her mother owned a sewing business. These wealthy roots and her extrovert personality, led María Luisa’s parents to school her under local French Nuns, allowing her to complete her bachelor studies in Torrelavega. In 1934, María Luisa Herreros moved to Madrid to study at the Medical School of the *Universidad Central de Madrid* (now the *Universidad Complutense*), which was relatively exceptional for Spanish women at that time^9^. She lived at the *Residencia de Señoritas* (see above), an institution founded by the *JAE* in 1915, 5 years after its male counterpart. This unique institution was founded thanks to generous support from the United States to promote higher education among Spanish women (involving the *Boston Committee* and an active exchange program with *Smith College*). Indeed, the *Residencia de Señoritas* occupied the former *International Institute for Girls in Spain*, owned by the United States government (Pérez-Villanueva Tovar, 2011). The *Residencia de Señoritas* was home to women over 16 years old who were officially studying or aspiring to be admitted to university, the *Higher School for Magisterium*, the *National Music Conservatory*, the *Normal School* or similar institutions. Although forward looking from an academic point of view, the internal regime was a mirror of the times, including *“the freedom of a well-organized Spanish family, including diligent attention and meticulous surveillance”* (Capel Martínez, 2009).

The Director of the *Residencia*, María de Maeztu Whitney (1882–1948), was an important educator and feminist activist from a well-known intellectual family. Her brother Ramiro was a right-wing reformist thinker, writer, journalist and diplomat, and her other brother, Gustavo, was a painter. The international element in the Maeztu family came from her father, a civil engineer born in Spanish Cuba (see above), where he worked and married the daughter of a British diplomat. In the internal files of the *Residencia de Señoritas*, María Luisa Herreros was reportedly extremely interested in her classes, and she was open and nice. Maria Luisa learnt Histology and Pathology from Prof. Jorge Francisco Tello, not only the first true disciple of Cajal but also, his successor as university chair and as director of the *Instituto Cajal* until 1939. Her Physiology professor was Juan Negrín (1892–1956), who became the last prime minister of the IInd Spanish Republic (1937–1939). Both these professors gave María Luisa Herreros the highest grades, as seen in the academic records from the *Universidad Central*, Madrid, and in those of the *Residencia de Señoritas*.

But the political situation in Madrid at the end of the 1935–1936 academic course was very tense, with the frequent shooting of activists on the far-left and far-right. As a result, María Luisa decided to go back home, close to the Cantabrian seaside, which is where she was at the outbreak of the Spanish Civil War. The province of Santander (currently the autonomic region of Cantabria) was initially part of the area controlled by the legal Republican government. María went to work at the military hospital that opened in Torrelavega and she also helped perform autopsies on those who were assassinated by being thrown from the Santander lighthouse into the wild sea. The war ended for her in August 1937, when the rebel troops of general Franco occupied this Northern region.

When the Spanish Civil War ended (1939), María Luisa Herreros returned to Madrid to continue her studies in Medicine. She again lived in the pavilions of the former *Residencia de Señoritas*, now transformed into the *Colegio Mayor Teresa de Cepeda* and later, into the *Colegio Mayor femenino Santa Teresa de Jesús* due to the dissolution of the *JAE* by Franco. Although the general and political situation in Spain had changed, the new Director of the *Colegio Mayor Santa Teresa de Jesús* was Matilde Marquina, who tried to continue ensuring that women had access to higher education, adding *“special attention to the religious and moral education of the residents”* that included daily religious services in the chapel (Marquina, 1945). As can be read in her academic records (Universidad Central, Madrid), Herreros was allowed to continue with her university studies by declaring that she *“had not collaborated with the governments of the Popular Front”*. She also had to become affiliated to the *Sindicato de Estudiantes Universitarios-SEU*, the only legal student organization at that time in Spain, which was aligned with the fascist party *Falange Española Tradicionalista y de las J.O.N.S.^10^*, the only political party officially allowed during the dictatorship (1939–1975).

**FIGURE 6:**
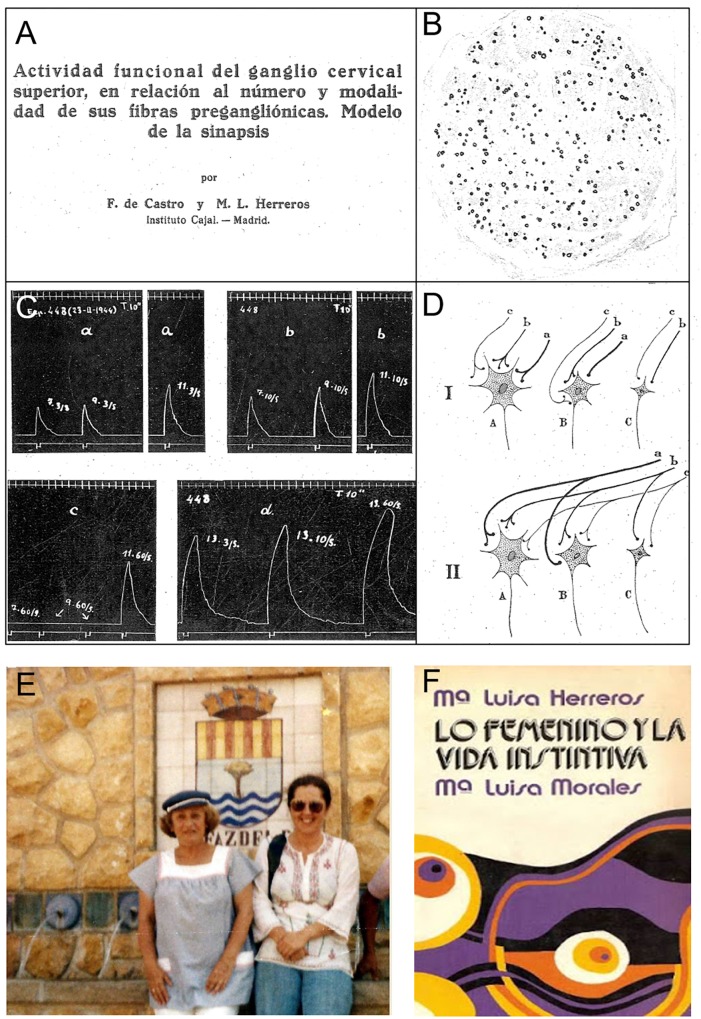
Dr. María Luisa Herreros. **(A)** Heading of the article published under the direction of Dr. Fernando de Castro at the *Cajal Institute* (de Castro and Herreros (1945)). **(B)** Microphotograph from a histological section of the sympathetic trunk of a cat in which the axons from all the thoracic rami but the IIIrd were experimentally sectioned 58 days previously. Myelin is stained with osmic acid (originally published as Figure 18 in de Castro and Herreros (1945)). **(C)** Electromyograms of the nictitating membrane in response to stimulation of the sympathetic trunk, obtained from a cat in which all the ipsilateral sympathetic thoracic rami but the Ist were previously sectioned (originally published as Figure 21 in de Castro and Herreros (1945)). **(D)** Diagram illustrating the convergence of the preganglionic fibers (*a–c*) onto the three different types of ganglionic cells identified (*A*–*C*), and a scheme of the thickness of the different afferents, as well as the need to activate more than one synapse to trigger postsynaptic responses. This scheme was originally published as Figure 28 in de Castro and Herreros (1945). **(E)** On the left, Dr. $M^{\underline{a}}$ Luisa Herreros ca. 1973 at El Alfaz del Pí, a well-known spa on the coast of Alicante (Spain), where she bought an ancient mill that she restored and used as second residence for holidays (originally published by Aramburu at her blog: http://psicologos-benidorm.blogspot.com/2015/12/la-doctora-maria-luisa-herreros-y-yo.html). **(F)** Front page of the book “Lo femenino y la vida Instintiva,” published by Dr. Herreros and her colleague Dr. Morales in 1973 (Herreros and Morales, 1973).

In 1943, María Luisa Herreros obtained her M.D. at the Universidad Central de Madrid and she began her doctorate studies there, focusing on Neuroscience and Endocrinology. It was then when she worked at the *Instituto Cajal* with Fernando de Castro (see above), one of the few researchers from the Spanish Neurological School who remained in Madrid throughout the Spanish Civil War, defending the building together with Tello (de Castro, 1981, 2019b; Vial, 1996; De Carlos and Pedraza, 2014)^11^. Herreros and de Castro studied the structure and function of synapses in the superior cervical ganglion (de Castro and Herreros, 1945) (Figure 6A–D). They showed that there is no segmental distribution of sympathetic innervation and that preganglionic axons are distributed throughout the ganglia without cellular preference: the only direct correlation being in the amount of terminal boutons and the bulk of the afferent fibers. As a final conclusion, de Castro and Herreros suggested that the pattern of synapses in the sympathetic ganglia is of the *“diffuse type, similar to that in the molecular layer of the cerebellum”*, as described previously by de Castro (1942) and unlike the “circumscript type” found in the spinal cord nuclei, the brainstem and other parts of the brain (Figure 6D). Curiously, this is the first scientific paper partially written by Fernando de Castro in English (it includes a Summary in English at the end of the article), for which the authors thank Dr. Francisco Grande-Covián, who corrected and improved the English text. We can also see here de Castro’s erroneous conception of the nature of the synapse/synaptic cleft (a physical interposition of glial processes between the pre- and post-synaptic ends)^12^.

Subsequently, Dr. Herreros branched out into another field of Neuroscience, Psychiatry, and she registered as “research physician” with the *Official College of Surgeons of Cantabria* in 1948, only the third woman registered by this institution in that Province at that time. María Luisa Herrero then volunteered to join the research group of Dr. Gregorio Marañón^13^, extremely famous in Spain, at the *Instituto de Patología Médica* in the *Hospital Provincial de Madrid*, where from the outset she began working in Neuropsychiatry (Herreros, 1953c). Dr. Herreros performed psychodynamic studies on patients with thyroid pathologies, publishing two scientific papers on the psychic etiology of Basedow’s disease and on the simple goiter that gives rise to hyperthyroidism (Herreros, 1953a,b). In these studies, psychic factors were considered as fundamental etiological agents, recommending psychoanalysis as a complementary treatment to the endocrine therapies.

Dr. Herreros became interested in psychoanalysis from her beginnings as a psychiatrist, even though, for different reasons, this therapeutic approach was not very popular among medical professionals in Spain. Indeed, and perhaps most importantly, Santiago Ramón y Cajal had a very strong opinion of psychoanalysis and in the words of José Lázaro, Cajal thought that *“… the basis of mental diseases must reside in morphological changes in the brain”* (Lázaro, 2000; López-Muñoz et al., 2008). In addition, the efforts of Ángel Garma [the first psychoanalyst in Spain recognized by the *International Psychiatry Association* (*IPA*)] to implement psychoanalysis in Spain were halted abruptly by the outbreak of the Spanish Civil War. Indeed, the authoritarian regime established in Spain after the end of the Civil War was firmly based on rigid traditionalism that admitted no discrepancies, inculcating religious and military values that were not propitious for the development of psychotherapies. Nevertheless, Jerónimo Molina Núñez, a disciple of Garma, tried to keep the psychoanalytical flame alight and he found a way for Margarita Steinbach (an analyst from the recently re-established *Deutsche Psychoanalytische Verbindung*) to come to Spain in 1950. Steinbach worked actively in setting up the first group of psychoanalysts in Madrid and she submitted María Luisa Herreros to a psychoanalysis. Subsequently, Molina Núñez, Ramón de Portillo, María Teresa Ruiz and María Luisa Herreros founded the *Asociación Española de Psicoanálisis-(AEP)*, officially approved and registered in 1954. In order to become recognized by the IPA, a number of psychoanalysts from Madrid and Barcelona attended a meeting in London (United Kingdom), where María Luisa Herreros and Teresa Ruiz met Anna Freud (the daughter of Sigmund Freud). Mrs. Freud invited these Spaniards to tea at her own home and she advised them how to get the embryonic Spanish association (*AEP*) accepted as a member of IPA. In July 1957, at the 20th meeting of the *IPA*, it was agreed to assess the Hispano-Portuguese group of psychoanalysis and finally, the *Sociedad Española de Psicoanálisis (SEP)* was accepted as a formal member of the *IPA* in 1959. From the very moment that she became interested in psychoanalysis, Dr. Herreros remained strongly influenced by Freud, although she never occulted her fascination for Carl-Gustav Jung As such, Herrero’s therapeutic approaches could not be circumscribed to a single school.

Dr. Herrero’s career as a clinical psychoanalyst was intense and extensive, publishing the chapter entitled “Norms for Psychotherapy” in a very famous textbook by Prof Juan Rof-Carballo (Rof-Carballo, 1954). In 1973, and together with her disciple María Luisa Morales, Dr. Herreros published a book on feminine issues and instincts, a work that is still considered a force in the treatment of sexuality, the relevance of transcendent love, the implications of the conscience and inconscience in the game of love, transgressions and mental health (Figure 6E,F; Herreros and Morales, 1973). Maybe the main question in this book is what is at the heart of being feminine for it to be historically repudiated by our society. Jung, a key figure in the early days of psychoanalysis, considered that there are both masculine (“animus”) and feminine (“anima”) components within every human being, independently of her/his gender: the feminine aspects are associated to care, reception, protection, feeling, intuition, tenderness and empathy, all aspects that move men to have the disturbing sensation of losing control, giving rise to concern and a rejection of those sensations (Saiz Galdós et al., 2007). All these facets have been classically linked to women, both by men and society, and repudiating these aspects triggers a rejection of women.

Together with some collaborators (Gloria Enríquez de Salamanca, María Luisa Morales and Maite del Moral Sagarminaga), Dr. Herreos funded *Psique* In 1976, an association for research into and the application of psychoanalytic therapy, and mainly, to train new generations of psychoanalysts. However, the project was cut short by the Hogdkin’s lymphoma that caused María Luisa Herreros’ death in Madrid on October 3rd, 1985. This was the second death within the Spanish Neurological School due to Hodgkin’s lymphoma, which also caused the premature death of one of Cajal’s direct disciples in 1918, Nicolás Achúcarro (de Castro, 1981). In Spain, María Luisa Herreros was a pioneer in a masculine world in which higher Education, Culture and Science were almost exclusively the domain of men. She lived through a war and its consequences, and her rich scientific career commenced in the world of Histology following her University studies, and it moved into Psychiatry and psychoanalysis, a discipline where she shone until the end of her days.

## Conchita Del Valle and the Other Illustrators Collaborating With the Researchers of the Spanish Neurological School

Although chronologically this section represents a step backward, this work would not be complete without mentioning and briefly analyzing the role of the women illustrators that collaborated with the Spanish Neurological School. Although microphotography began to be used in Cajal’s lab at the beginning of the 1920s, it could not compete with the quality and quantity of information gained from the histological drawings of the stained material. This was usual at the time until new staining procedures were developed that generated less background, and the situation also changed dramatically in the 1970s with the development of fluorochromes, and of antibodies conjugated with these for immunohistochemistry and immunocytochemistry. However, it is true that after the II World War, scientific photography became more and more common and manual drawing was phased out from laboratories, and hence, from the scientific literature. But drawing by hand was undoubtedly the perfect complement to the Golgi method and to the other simple techniques on which modern Neuroscience was founded. Besides being ground-breaking scientists, Cajal and some of his disciples, like his brother Pedro, Domingo Sánchez, Achúcarro, del Río-Hortega, de Castro and Lorente de Nó, were true masters of this art (de Castro, 1981; DeFelipe, 2017). Yet curiously, one of Cajal’s main disciples, Francisco Tello, was not talented in illustrating his observations and therefore, he requested the technical help of illustrators, almost all of whom were women.

**FIGURE 7:**
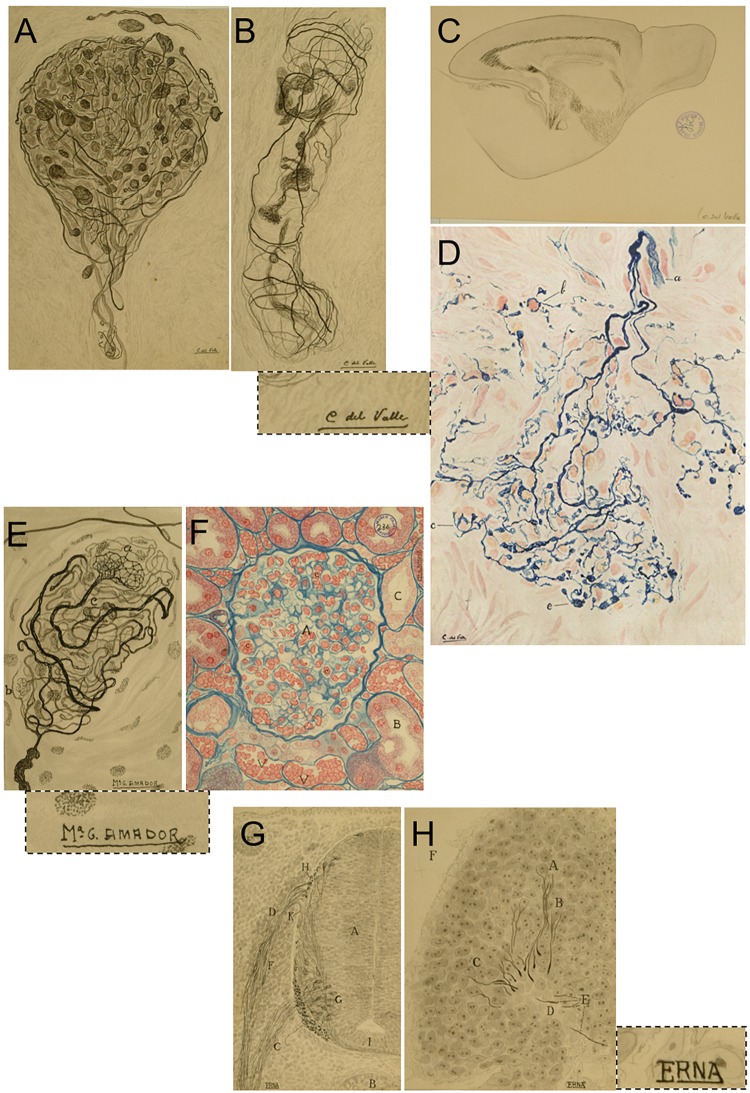
Conchita del Valle and other illustrators working with Francisco Tello. **(A–D)** Original drawings signed by Conchita del Valle that illustrate details of the sensory terminals in the clitoris. **(A,B)**, A detailed reproduction of a sagittal section from the brain of a 20 mm long mouse **(C)** and her only known polychrome drawing illustrating details of the sensitive innervation of the carotid body **(D)**, originally published in de Castro (1940). This latter original drawing from Conchita del Valle belongs to the *Archivo Fernando de Castro* (Censo–Guía de Archivos de España e Iberoamérica #ES.28079.AFC; Madrid, Spain), that in 2017 was included by *UNESCO* in the Memory of the World International Register of the Human Heritage, as “Archives of Santiago Ramón y Cajal and the Spanish Neurohistological School” (http://www.unesco.org/new/en/communication-and-information/memory-of-the-world/register/full-list-of-registered-heritage/registered-heritage-page-1/archives-of-santiago-ramon-y-cajal-and-the-spanish-neurohistological-school/). Dotted line, a detail of the signature “C. del Valle” in **C**. **(E**,**F)** Examples of the original drawings signed by $M^{\underline{a}}$ G. Amador, illustrating the sensory terminals of the clitoris (**E**; this drawing was maybe used for the same work as **A**,**B**) and one of her polychrome illustrations showing the structure of the kidney stained by the Heidenhan’s azan method. Dotted line, a detail of the signature “$M^{\underline{a}}$ G. Amador” in **E**. **(G,H)** Two of the drawings signed by E.RNA, illustrating details of the chicken embryo after 72 (**G**, which shows a transverse section of the spinal cord and a somite) or 40 h incubation **(H)**. Dotted line, a detail of the signature “E.RNA” in **H**. **(A–C,E–H)** Belong to the *Legado Cajal*, and they are published here with the permission and courtesy of the *Cajal Institute, Cajal Legacy, Spanish National Research Council (CSIC)*, Madrid, Spain.

Having studied all the original illustrations from Tello’s neurohistological works^14^ (part of which can be found in the *Legado Cajal*, conserved at the *Instituto Cajal* since the death of *Don Santiago*), we conclude that: 84 of these were signed by “Del Valle” or “C. del Valle,” referring to Conchita del Valle; 71 by “$M^{\underline{a}}$ G. Amador”; 141 by “ERNA” (or “E.RNA”); and 247 are not signed (although 4 of them are likely to be produced by C. del Valle due to their style and subject matter). There is little information about these illustrators, although the most notorious and well-known is undoubtedly Conchita del Valle, because of her fine attention to detail and artistic (yet realistic) composition (Figure 7A–D). A subtle detail that is perhaps proof of the relevance of Mrs. del Valle as an illustrator, she is the only one among the names cited above that can be identified in the *Legado Cajal^15^*, where 8 photomechanical prints (printing proofs) indicate from “drawings of C. del Valle from microphotographs,” although only two of them are signed by “C. del Valle”. While it was Francisco Tello who most specifically needed this technical assistance, the illustrators eventually collaborated with other researchers as well. One of the most distinguished neuroscientist in the laboratory, Fernando de Castro, despite his own talent as an artist, once requested the collaboration of Conchita del Valle to specifically take advantage of her talent as an illustrator to depict one of the terminals at the carotid sinus after a 12 day ablation of the sympathetic trunk. This was a polychrome image used in de Castro’s first paper published after the Spanish Civil War, and once Heymans had been awarded the Nobel Prize (Figure 7D; de Castro, 1940; for a modern review on the race to reveal the nature of arterial chemoreceptors between Heymans and de Castro, see: de Castro, 2009). This is an extremely rare exception in de Castro’s works, where all the illustrations were usually original works by the author^16^, and it reflects the respect Fernando de Castro had for the quality of the drawings produced by Conchita del Valle.

Regarding Mrs. Amador, it should be noted that “$M^{\underline{a}}$” is an abbreviation of the name “María” in Spanish (Figure 7E,F). On the other hand (Figure 7G,H), there is no proof that the signature “ERNA” or “E.RNA” was a woman and while we assume this to be the case, we cannot be 100% sure. It can be concluded that of the original drawings attributed to Francisco Tello and conserved at the *Legado Cajal*, more than 50% are signed by these three illustrators. Conchita del Valle illustrated almost exclusively structures in the CNS or the PNS, and we should highlight two special series among her drawings: one devoted to the innervation of the clitoris (just 2 drawings of this series are signed by $M^{\underline{a}}$ G. Amador); and a fantastic series of sagittal sections of the neonatal/early postnatal mouse brain. ERNA (or E.RNA) almost exclusively produced illustrations related to mouse and chick embryo development, and to a lesser extent, early postnatal development. Del Valle and Amador also signed some drawings referred to simply as “mouse embryo.” Only 12 of the drawings attributed to Francisco Tello are polychromes: 10 of them are signed by $M^{\underline{a}}$ G. Amador, one by C. del Valle, and the last one is unsigned. Finally, it should be noted that the unsigned drawings include those related to some of Tello’s most relevant contributions, and they are experiencing a kind of revival in modern times: illustrations of the innervation of the motor plates and the regeneration of peripheral nerves (Tello, 1905, 1907, 1914, 1917).

Unfortunately, no more information is available about these women, and we cannot identify the name of any of them with any kind of guarantee in the group pictures of Cajal’s School (see Figure 1B for an example of these group pictures). A more detailed study might help to attribute authorship to more of these drawings, as well dating them, which could help the future identification of these relevant neuro illustrators.

## The Case of the Influential Librarian

We finish the list of the women of the Spanish Neurological School by considering the first librarians at the Cajal Institute, the sisters Irene and Enriqueta “Ketty” Lewy. This was a “particular case” in the scope of the present work because neither of them can be considered researchers. After the publication of her first book (Rodríguez, 1977), Ketty Lewy claimed to have been the secretary of Cajal and she insinuated that no other women within the circle of the maestro had a more important role than she; her testimonies contributed to idea that the Cajal School was “free of women.” Mrs. Enriqueta Lewy (1910–2001; Figure 3A) joined the *Instituto Cajal* as librarian when she was still a girl (only 16 years old), substituting her sister, Irene Falcón^17^, when she moved to London in 1926. Both sisters were born in Madrid to a Polish middle-class businessman, Siegfried Lewy, and they were raised as German speakers before Mr. Lewy abandoned his family. This aspect of Ketty’s education was useful for Cajal and other researchers to translate works published in German, and especially, to write letters to and communicate with German-speaking scientists. Yet Mrs. Lewy was a very secondary actor in Cajal’s circle (Pérez, 1929; de Castro, 1981), her importance waning even before her political exile to the USSR and the Popular Republic of China for 20 years after the Spanish Civil War^18^. Despite her evident communist links and her activity in exile (she worked in political spaces at the official communist radios in both countries), the former librarian had no problems to return to the Spain of Franco in 1971 and she was hired by the Spanish Research Council (*Consejo Superior de Investigaciones Científicas-CSIC*), the public administration that Franco founded in 1939 to absorb the *Instituto Cajal* and to take on the other responsibilities of the *JAE*. There, she worked in the Scientific Documentation Service and she collaborated with the journal *Arbor*, published by the *CSIC* (Falcón, 1996). Only a decade after the death of the penultimate direct disciple of Cajal, Fernando de Castro (see above), Mrs. Lewy published a book about *“her life with Cajal”* that gained the attention of Spanish devotees of Cajal, although a huge part of the text was simply a transcription of Cajal’s own memoirs, press articles or speeches (Rodríguez, 1977^19^). True experts consider this book more a kind of “auto-hagiography,” full of imprecisions and errors. For example, the reference to the death of Cajal clearly clashed with the published press reports and multiple testimonies from those who were present when Santiago Ramón y Cajal died (de Castro, 1981; Grande Covián, 1984). In the last 20 years of her life, Ketty Lewy exploited the efficient networks of the far left in the newly democratic Spain to spread her views on Santiago Ramón y Cajal, his scientific disciples, his intellectual circle and even his family. In this way she carefully spread the idea that she was the only relevant woman at the Cajal Institute, and she was fundamental in relegating to the shadows the truly relevant women in this story, the subject of our present work^20^. As the sister of a relevant feminist activist and claiming to be a feminist activist herself (Niño, 1996), it is difficult to understand why Mrs. Lewy didn’t even mention the women with whom she was pictured at the *Cajal Institute* (Figure 1B). Indeed, it is noteworthy that she published her famous “auto-hagiography” when the last direct disciples of Cajal had either died (Fernando de Castro) or retired to the sunny but distant California (Rafael Lorente de Nó, when 75 years old). It is also remarkable that during her exile in China, she wrote to Fernando de Castro proposing to translate the technical manual he produced with Cajal (Cajal and de Castro, 1933) into Chinese, fully sponsored by the Chinese Army (letter conserved at the *Archivo Fernando de Castro*, Madrid, Spain^21^). The diffusion of Lewy’s book (Rodríguez, 1977) pushed the silenced figures of the female neuroscientists working in the Cajal School further into the shade. Here, we wanted to again highlight their names and achievements, giving them the recognition they truly deserve.

## Discussion

We present here the brief biographies and the scientific achievements of four women who carried out at least part of their research with Santiago Ramón y Cajal or other important members of the Spanish Neurological School, such as Fernando de Castro or Gonzalo R. Lafora. Astonishingly, this facet of the School has remained largely ignored, even though three of them published scientific articles in the journal founded by Cajal. Cajal himself included the first two (Laura Forster and Manuela Serra) in the list of his School depicted in 1922 (Table 1), and included in Ramón y Cajal (1923). We also wanted to highlight the relevant contribution of women to the support staff at the institute, like the illustrators that helped produce the scientific publications from the Spanish Neurological School, mainly supporting Jorge Francisco Tello.

It is remarkable that the first woman documented here was a British/Australian scientist, Laura Forster, who came to Madrid before the 1st World War in which she enrolled to serve her country. Dr. Forster’s brilliant public career in Science and Health, as highlighted here, was cut short by her death while directing a field–hospital at the Russian front. Dr. Herreros career was also outstanding and she became one of the founders of psychoanalysis in Spain, while Dr. Ruiz-Capillas, career in healthcare was no less important. The life of Manuela Serra remains virtually undocumented. Besides what we present here, no other traces of her could be found, neither at the *Instituto Cajal*, the *Spanish Research Council* (*JAE* Archives) nor in the University archives. Some experts confused her with her sister, Mrs. Carmen Serra, who was also a laboratory assistant (named by Cajal and his disciples “preparadora” as they were specialists in performing histological preparations) who worked for years at the *Instituto Cajal*, perhaps better known to experts in Cajal’s circle due to her presence in different photographs (Figure 1B, 3A; de Castro, 1981; Carmen Serra is identified by name in seven photographic plates of the Legado Cajal). These women, as well as Conchita del Valle and the other neuro illustrators deserve the public recognition that has been denied them for decades. What is particularly notable and surprising is the lack of references to these women by one of their colleagues, Ketty Lewy (Figure 3A), especially given her links to the feminist movement and because she supposedly intended to accurately reflect Cajal’s circle of colleagues and acquaintances in his latter years in her book.

**FIGURE 8:**
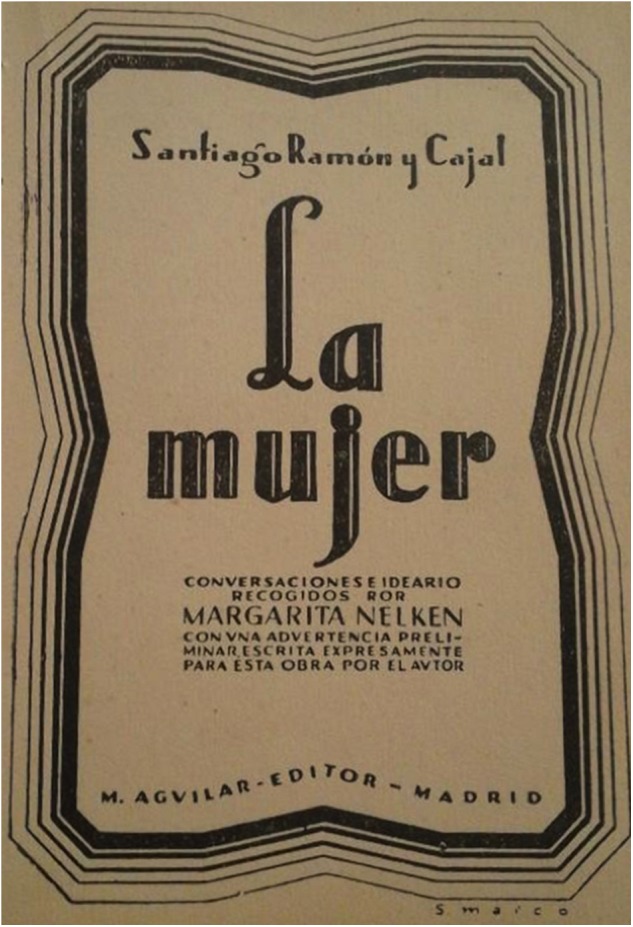
Front–page of the book “La mujer” (“The woman”), including texts from Santiago Ramón y Cajal and his conversations with Margarita Nelken (Ramón y Cajal, 1932).

But while it may fit with the times that the first women researcher at the Cajal School was a British/Australian, the others were all Spaniards. This sheds some doubt on a *cliché* regarding the delay of women’s incorporation into Spanish academia. In parallel, there is a general assumption that Cajal was a male chauvinist, mainly derived from chapter VI in his universally famous book *“Reglas y consejos sobre investigación biológica”*. This text was conceived as Cajal’s acceptance speech when entering the Spanish National Academy of Medicine, written in 1897 and later published as a book that has been translated into many languages, from English to Japanese (Ramón y Cajal, 1899). In this particular chapter, there are different recommendations and judgements about the wives of scientists that we will not go into in detail here. However, Cajal’s posture modified in his later years during which Cajal spoke out and wrote against the inferiority of women vs. men. In response to the known Spanish politician and feminist activist, Margarita Nelken, in 1925 Santiago Ramón y Cajal wrote: *“It is strange what happens to me with militant feminists. They only read those authors who whip them, wrapping their criticisms with an overwhelming scientific or pseudoscientific rhetoric, well wrapped in polite and sweet phrases. And on the other hand, the few who, in defying the wrath of misogynists, have defended women from the biological and from other points of view, we have not deserved the honour of being mentioned for those passages of our books favourable to their just demands”* (Fernández Santarén, 2014). Mrs. Nelken had previously proposed Cajal put together all his opinions about women (from biology to education) in a book that was finally published 7 years later and under an unequivocal title: *“The Woman”* (Figure 8; Ramón y Cajal, 1932). It is unclear why this important book is still ignored by academics pontificating and writing on the subject of Cajal, and on his thoughts about women in the most prestigious environments. They simply focus on the old anecdotes and comments, perpetuating the image of Cajal as a male chauvinist (e.g., Cruz Hermida, 2006). As such, we deduce that many of the commentaries and personal anecdotes (some of them mere capers) regarding Cajal were more just a reflection of the society and times in which he lived. In that environment, women who entered Science in the late 19th and the start of the 20th century were undoubtedly extraordinary. Whether Cajal changed his mind about women as scientist or not is irrelevant, what is important is how women like Laura Forster and others contributed to his hypothetical metamorphosis, as confirmed on his own writing in the aforementioned book (Ramón y Cajal, 1932). Here, we want to emphasize that Santiago Ramón y Cajal was open to accept women and work with them, and not only as secondary collaborators (lab assistants, illustrators, librarians) but also as independent researchers, going as far as including two of them in his own depiction of his scientific school in 1922 (Table 1; Pérez, 1929).

The role of women neuroscientists within the Cajal School was perhaps not as prominent as that of the female neuroscientists in other countries: the Russians Maria Manasseina (1841–1903), who worked with Ivan Tarkhanov on sleep research and pioneered studies into the effects of sleep deprivation on animals, and Lina Solomonovna Shtern (1878–1968), one of the pioneers on the blood-brain barrier; the Polish-born Micheline Stefanowska (1855–1942), who worked in Switzerland, Belgium and France, studying a variety of issues from pain psycho-physiology to dendritic spines – she described the plasticity of dendritic spines after electric stimulation, a hypothesis that Cajal, who first described these structures in 1888, considered plausible but not fully demonstrated at that time; the American-born French clinician Augusta Marie Déjerine-Klumpke (1859–1927), first woman to work as an intern in a hospital in Paris and who described Klumpke palsy caused by damage to the peripheral nerves controlling arm movements, author of about 60 papers and co-author with her husband, Joseph Jules Déjérine, (1849–1917) of the two-volume book “Anatomie des Centres Nerveux,” both disciples of Vulpian; the French Cécile Vogt (1875–1962, born Cécile Mugnier), disciple of Pierre Marie, who devoted special attention to the study of myelination and the white matter of the brain, and who was as relevant as her husband^22^, Oskar Vogt (disciple of Déjerine and Déjerine-Klumpke), for decades both leading the work of the so-called “brain localizationists” in Germany (including famous scientists, like Korbinian Brodmann or Max Bielschowsky); the Russian-born Marie Nageotte-Wilbouchewitch (1864–1941), M.D. from the *Université de Paris* (France), who collaborated with her husband Jean Nageotte on the study of neuroanatomy and different CNS pathologies, a recognized Pediatrician who became the first president (of any gender) of the *Societé Française de Pédiatrie*; the Romanian-born French psychiatrist Constanza Pascal (1877–1937), specialist and pioneer in dementia and dementia praecox; the German medical doctor Martha Ulrich (1881–1943), maybe the first woman to ever publish an article on glial cells; and undoubtedly the most influential and well-known, although corresponding to a later generation, Rita Levi-Montalcini (1909–2012), a graduate in Giuseppe Levi’s laboratory when Fernando de Castro worked at Torino in 1934, who obtained the Nobel Prize in 1986 with Stanley Cohen after decades of international recognition for the discovery of the Nerve Growth Factor (Stefanowska, 1897; Ioteyko and Stefanowska, 1909; Ulrich, 1910; Levi-Montalcini, 1987; van Gijn, 2003; de Castro, 2009, 2016; Sierra et al., 2016; DeFelipe, 2017; Favero et al., 2017; Metitieri et al., 2017; Metitieri and Mele, 2018). Nevertheless, the contributions of women in Cajal’s laboratory was not dissimilar to that in other fields of Spanish experimental sciences, where the presence of women has been studied in more depth (Magallón, 2007). Interestingly, Cajal indicated that some of these neuroscientists (Mrs. Déjerine, Nageotte and Vogt, besides Mme Curie) were examples of the type of woman that he would like to marry (Ramón y Cajal, 1899).

## Conclusion

Although female researchers were little known within the School of Cajal, the laboratory was open to accepting women and not only as secondary collaborators (lab assistants, illustrators, librarians) but also as independent researchers, to the extent of including two of them in his own description of the school in 1922 (Table 1; Ramón y Cajal, 1923; Pérez, 1929). We hope that in this article we have been able to give an accurate biography of these extraordinary women, helping them to gain greater recognition for their scientific contributions, as well as offering a more complete reflection of the attitudes toward gender in what was perhaps one of the most fruitful scientific schools in the field of Biomedicine worldwide: the Spanish Neurological School.

## Author Contributions

EG proposed the study. FdC, CN, and EG performed the research. FdC, CN, and EG wrote the manuscript. FdC prepared the figures. EG, CN, CM, and CS corrected the manuscript and contributed with figures for some panels.

## Conflict of Interest Statement

The authors declare that the research was conducted in the absence of any commercial or financial relationships that could be construed as a potential conflict of interest. The handling Editor and reviewer J-GB declared their involvement as co-editors in the Research Topic and confirm the absence of any other collaboration.

## Footnotes

1 The word “neurone” or “neuron” was coined by Heinrich Wilhelm Gottfried von Waldeyer-Hartz (1891) in an article published in 1891 (Waldeyer-Hartz, 1891). Although a relevant anatomist, Cajal himself pointed out that “Professor Waldeyer, to whom poorly informed persons attribute the neuron theory, supported it with the prestige of his authority, but did not contribute a single personal observation. He limited himself to a short, brilliant exposition of the objective proofs, adduced by His, Kölliker, Retzius, van Gehuchten and myself, and he invented the fortunate term of ‘neuron”’ (Ramón y Cajal, 1933; the translation corresponds to the English version by Drs. M. Úbeda Purkiss and Clement A. Fox, published in 1952 by the Spanish Research Council-CSIC, Madrid, Spain).

2 Originally known as *Laboratorio de Investigaciones Biológicas*, the laboratory of Cajal officially became the *Instituto Cajal* in 1920 (De Carlos and Pedraza, 2014).

3 Prof. Francis Gotch (1853–1913) was the direct predecessor of Charles S. Sherrington (Nobel prize winner in Physiology or Medicine, in 1932) as head of the *Physiological Laboratory* at the *University of Oxford*. This was in 1913, yet long before, in 1895, Sherrington got his first full-professorship as Holt Professor of Physiology at the *University of Liverpool*, succeeding Francis Gotch. This first Gotch-Sherrington succession was especially important for the latter, who left pathology to become one of the most important physiologists in the History of Neuroscience (Eccles and Gibson, 1979).

4 Other disciples of Cajal who were universally recognized as masters of neurohistological illustration were Pío del Río-Hortega, Fernando de Castro and Rafael Lorente de Nó, yet all them arrived at Cajal’s lab after Laura Forster had left.

5 Translated from the original in Spanish by the authors of the current work.

6 http://cedros.residencia.csic.es/imagenes/Portal/ArchivoJAE/memorias/009.pdf

7 Microglial cells were first described by Pío del Río-Hortega (del Río-Hortega, 1919a,b,c; del Río-Hortega, 1920; these papers have recently been translated into English in: Sierra et al., 2016).

8 The original description in Spanish of these mitotic cells deserves to be reproduced here for those Spanish speaking readers: *“… corpúsculo en vías de mitosis (fase de* estrella madre*), cuyo soma, más o menos redondeado, exhibía en su porción cortical diversas gliofibrillas dispuestas en remolino y trazando eses, ochos de guarismo y otras curvas complicadas”.*

9 At the beginning of the 1920s, Spanish universities had just 21 official female alumni, while there were 29 women at the Superior School for primary school teachers.

10 The *Falange Española Tradicionalista y de las J.O.N.S.* resulted from the fusion of the original *Falange Española de las J.O.N.S.* (itself a mixture of fascists and revolutionary pseudo-fascists) and the *Comunión Tradicionalista Carlista* (supporters of a traditional absolutist monarchy, ultraconservative and old-fashioned Catholics). This fusion was as artificial as it was traumatic for both parties, resulting in a very difficult political coexistence.

11 Both Tello and de Castro were expelled from the University but they were allowed to continue working at the Instituto Cajal in poor conditions, and with no responsibilities at all (de Castro, 1981; Vial, 1996; De Carlos and Pedraza, 2014).

12 Some authors have linked this erroneous glial interposition to our current vision of synaptic plasticity or one of the most modern revolutionary concepts in Neuroscience, the “tripartite synapses” described at the end of the 1990s as the basis of the intervention of astrocytes in synaptic transmission (García-Segura, 2002; Perea et al., 2009; de Castro, 2016).

13 Gregorio Marañón (1887–1960), famous clinician, writer and liberal politician. Together with the philosopher José Ortega y Gasset and the author, Ramon Pérez de Ayala, Marañón, he was one of the founders of the “Agrupación al Servicio la República,” a collective of intellectuals and politicians that was determinant in the fall of the King Alphonse XIII and the advent of the IInd Republic in 1931. Indeed, it was in Dr. Marañón’s house in Madrid (in the aristocratic Street, Serrano) where the agreements to found the new regime and the King‘s exile were signed on April 14th, 1931. Disgusted with the violent and pre-revolutionary developments at the start of the IInd Republic, and in fear for his own life in Madrid once Civil War broke out, Dr. Marañón went into exile in Paris (France). As he explained in January 1937 at a meeting of the pro-Republican French intellectuals: *“You don’t need to try very hard, my friends; listen to this: eighty eight percent of the lecturers in Madrid, Valencia and Barcelona (the three universities which, alongside Murcia’s, had stayed on the republican side) have been forced into exile abroad. And do you know why? Simply because they were afraid of being murdered by the reds (communists in Spain), even though many of the threatened intellectuals were thought to be left-wingers”.* Once back in Spain, in 1942 Dr. Marañón worked at the Hospital Provincial de Madrid (current Hospital Gregorio Marañón) and in 1946, he recovered the Chair of Endocrinology at the Medical School of the Universidad Central (Madrid). There, he continued his research in this medical area, while building an extremely famous and prosperous private medical practice.

14 The final published illustrations were sometimes only a part of the original drawing. To avoid any confusion due to this manipulation, we have analyzed only the original drawings and not the published figures.

15 http://www.cajal.csic.es/LegadoCajal/index.php/. Counts made on November 29th, 2018 by FdC.

16 Besides this drawing by del Valle, we have also found a scheme published in 1962 by de Castro that is signed “F. Blanco”: they are maybe the only exceptions in the 50 plus year long research career of Fernando de Castro.

17 Irene Falcón (Madrid, 1907 – Madrid, 1999), born Irene Levy Rodríguez, adopted the surname of her husband, the journalist César Falcón, whom she married in Edinburgh (United Kingdom) when underage. With time, she became personal secretary to Mrs. Dolores Ibárruri “Pasionaria,” one of the most famous and faithful Stalinists among the leaders of the Spanish Communist Party before, during and after the Spanish Civil War (Niño, 1996).

18 As another example, on page 299 of Egido and Montes (2018) we find: “Del Instituto Cajal de la JAE formaron parte Enriqueta Lewy Rodríguez, traductora y secretaria *de Santiago Ramón y Cajal, al finalizar la Guerra Civil se exilió a la Unión Soviética, con su hermana Irene Falcón, secretaria de Dolores Ibárruri, Pasionaria, Manuela Serra, Marina García Palomares, como preparadoras, Dolores Pantoja Jiménez y Concepción Tovar Rodríguez”* (textually, including syntactic mistakes, in Spanish), citing reference [14] Otero Carvajal and López Sánchez, (2012) La lucha por la modernidad. Las ciencias naturales y la Junta para Ampliación de Estudios. Ed.: CSIC-Residencia de Estudiantes, Madrid (Spain). It is difficult to understand how the presence of female scientists in Cajal’s laboratory (or the Instituto Cajal) should be so ignorantly presented… in a book devoted specifically to the subject of modernity (including the incorporation of women into Science) and published by the CSIC, the Spanish Research Council founded in 1939 and including from then the *Instituto Cajal* itself…! The authors did not consult the index of the journal published by Cajal (and the *Instituto*), or other sources used here (Cajal’s own depiction of his school of disciples; important Spanish newspapers of that times).

19 It is interesting that although more widely known as Enriqueta (or Ketty) Lewy, she used the surname of her mother for the publication of this and other books and articles. In all of them she signed “Enriqueta L. Rodríguez.”

20 As another example, in Egido and Montes (2018) we find: *“Del* Instituto Cajal *de la* JAE *formaron parte Enriqueta Lewy Rodríguez, traductora y secretaria de Santiago Ramón y Cajal, al finalizar la Guerra Civil se exilió a la Unión Soviética, con su hermana Irene Falcón, secretaria de Dolores Ibárruri, Pasionaria, Manuela Serra, Marina García Palomares, como preparadoras, Dolores Pantoja Jiménez y Concepción Tovar Rodríguez”* (textually, including syntactic mistakes in Spanish), citing reference [14] Otero Carvajal and López Sánchez, (2012) La lucha por la modernidad. Las ciencias naturales y la Junta de Ampliación de Estudios. Ed.: CSIC-Residencia de Estudiantes, Madrid (Spain). It is difficult to understand how the presence of female scientists at Cajal’s laboratory (or *Instituto Cajal*) could be presented with such ignorance. In a book specifically devoted to modernity (including the incorporation of women into Science) and published by the *CSIC* (the *Spanish Research Council*, founded in 1939 and including the *Instituto Cajal*), the authors did not consult the index of the journal published by Cajal (and the *Instituto*) or other sources that we have used in the current work (e.g., Cajal’s own depiction of his disciples or important Spanish newspapers of the times).

21 The *Archivo Fernando de Castro* is included in the Censo-Guía de Archivos de España e Iberoamérica with the reference number #ES.28079.AFC, and it has been included by *UNESCO* in the Memory of the World International Register of the Human Heritage, as “Archives of Santiago Ramón y Cajal and the Spanish Neurohistological School” (http://www.unesco.org/new/en/communication-and-information/memory-of-the-world/register/full-list-of-registered-heritage/registered-heritage-page-1/archives-of-santiago-ramon-y-cajal-and-the-spanish-neurohistological-school/).

22 In their abundant publications together, Cécile was normally the first author. Their daughters, Marthe (1903–2003) and Marguerite Vogt (1913–2007) became a relevant neuropharmacologist at Cambridge (United Kingdom) and virologist at The Salk Institute (United States), respectively, the latter working with the Nobel prize winner Renato Dulbecco.
